# Health Factors and Risk of All-Cause, Cardiovascular, and Coronary Heart Disease Mortality: Findings from the MONICA and HAPIEE Studies in Lithuania

**DOI:** 10.1371/journal.pone.0114283

**Published:** 2014-12-05

**Authors:** Abdonas Tamosiunas, Dalia Luksiene, Migle Baceviciene, Gailute Bernotiene, Ricardas Radisauskas, Vilija Malinauskiene, Daina Kranciukaite-Butylkiniene, Dalia Virviciute, Anne Peasey, Martin Bobak

**Affiliations:** 1 Institute of Cardiology, Academy of Medicine, Lithuanian University of Health Sciences, Kaunas, Lithuania; 2 Department of Epidemiology and Public Health, University College London, London, United Kingdom; Shanghai Institute of Hypertension, China

## Abstract

**Aims:**

This study investigated the trends and levels of the prevalence of health factors, and the association of all-cause and cardiovascular (CVD) mortality with healthy levels of combined risk factors among Lithuanian urban population.

**Methods:**

Data from five general population surveys in Kaunas, Lithuania, conducted between 1983 and 2008 were used. Healthy factors measured at baseline include non-smoking, normal weight, normal arterial blood pressure, normal level of total serum cholesterol, normal physical activity and normal level of fasting glucose. Among 9,209 men and women aged 45–64 (7,648 were free from coronary heart disease (CHD) and stroke at baseline), 1,219 death cases from any cause, 589 deaths from CVD, and 342 deaths from CHD occurred during follow up. Cox proportional hazards regression was used to estimate the association between health factors and mortality from all causes, CVD and CHD.

**Results:**

Between 1983 and 2008, the proportion of subjects with 6 healthy levels of risk factors was higher in 2006–2008 than in 1983–1984 (0.6% vs. 0.2%; p = 0.09), although there was a significant increase in fasting glucose and a decline in intermediate physical activity. Men and women with normal or intermediate levels of risk factors had significantly lower all-cause, CVD and CHD mortality risk than persons with high levels of risk factors. Subjects with 5–6 healthy factors had hazard ratio (HR) of CVD mortality 0.35 (95% confidence interval (CI) 0.15–0.83) compared to average risk in the whole population. The hazard ratio for CVD mortality risk was significant in men (HR 0.34, 95% CI 0.12–0.97) but not in women (HR 0.38, 95% CI 0.09–1.67).

**Conclusions:**

An inverse association of most healthy levels of cardiovascular risk factors with risk of all-cause and CVD mortality was observed in this urban population-based cohort. A greater number of cardiovascular health factors were related with significantly lower risk of CVD mortality, particularly among men.

## Introduction

Despite the decline in age-standardized death rates over the recent decades in high-income countries, cardiovascular diseases (CVD), including coronary heart disease (CHD) and stroke, and cancer continue to be the leading causes of morbidity and mortality in the United States, most Western and Eastern countries [1]–[3]. The reductions in mortality and morbidity due to CVD and other non-communicable diseases can be attributed to several factors, including improvements in modifiable risk factors [4], [5].

Recently, new data from several studies have demonstrated the benefits of favourable levels of modifiable risk factors, so called cardiovascular health factors, for all-cause death rates and CVD mortality or morbidity [6]–[8]. Several studies, mainly in the US, have shown that alarmingly few adults participating in cohort studies achieved the favourable levels of all 7 most frequently measured CVD risk factors: physically activity, normal blood pressure, glucose and total cholesterol levels, body weight, and healthy diet [6], [9], [10].

In Lithuania, CVD incidence and mortality rates among both women and men are higher than in western European countries [11], [12]. In 2010, the mortality rates from CHD were 429 per 100,000 men and 239 per 100,000 women in Lithuania, compared with 210 per 100,000 for men and 114 per 100,000 women in 27 countries of the European Union [13]. Epidemiological studies in Lithuanian population samples indicate a high prevalence of most lifestyle (smoking, overweight and obesity, unhealthy nutrition habits, physical inactivity) and other modifiable risk factors of CVD (hypercholesterolemia, arterial hypertension) [14], [15]. The prognostic value of these risk factors in the incidence and mortality from CHD, stroke and other non-communicable diseases in Lithuania has been demonstrated to be similar to other populations [16]–[18]. However, risk factors have been studied separately and their combined effects have not been assessed.

The aim of this study was to estimate the trend and levels of the prevalence of health factors, and to evaluate the risk of all-cause and CVD mortality in relation to healthy levels of combined risk factors among middle aged Lithuanian urban population.

## Methods

### Study sample

Data from the five studies were used in these analyses. The first three surveys of the Lithuanian Multinational Monitoring of Trends and Determinants in Cardiovascular Disease (MONICA) Programme were performed in 1983–1984, 1986–1987 and 1992–1993; the post-MONICA survey was conducted in 2001–2002 using the MONICA protocol, and the 2006–2008 Health, Alcohol and Psychosocial Factors in Eastern Europe (HAPIEE) study adopted measures very similar to the MONICA Project [19]. All these surveys were carried out in Kaunas, the second largest Lithuanian city (population 348,624 people). All five surveys examined random samples of men and women aged, stratified by gender and age, randomly selected from the Kaunas population register. The response rates were 70.2%, 69.6%, 58.6%, 62.4%, 58.1%, respectively. A total of 9,209 subjects (7,648 were free from CHD and stroke at baseline) aged 45–64 at baseline were available for statistical analysis. All respondents provided written informed consent. All five studies were approved by the Regional Biomedical Research Ethics Committee at the Lithuanian University of Health Sciences and the HAPIEE study (2006–2008) - also by the UCLH Research Ethics Committee Alpha at University College London, UK.

### Baseline health examination

In each survey, blood pressure (BP), weight, height, and biomarkers were measured using the same methodology. The information and variables determined using the questionnaire was based on identical or very similar questionnaires.

### Measurements

BP was measured two times using mercury sphygmomanometer and appropriately sized arm cuffs on the right arm. The initial measurement was performed after five minutes of rest on the right arm. After two minutes, the second measurement was made. The Korotkoff phase 1 (beginning of the sound) and the fifth phase of Korotkoff (disappearance of the sound) was recorded as systolic and diastolic BP. The mean of two readings was used. Hypertension was defined as mean systolic BP of at least 140 mm Hg or mean diastolic BP of at least 90 mm Hg, or both, and/or using antihypertensive medication in the last two weeks. Weight and height were measured with a calibrated medical scale, and without shoes or heavy clothes. Body mass index (BMI) was calculated as the weight in kilograms divided by the height in meters squared (kg/m^2^). Normal weight was defined as BMI<25.0 kg/m^2^, overweight as BMI≥25.0–29.99 kg/m^2^ and obesity as BMI≥30.0 kg/m^2^.

### Laboratory analyses

Fasting serum samples from first four surveys were analyzed in the Laboratory of the Institute of Cardiology of the Lithuanian University of Health Sciences; serum samples from the fifth survey were analysed in one batch in the WHO Regional Lipid Reference Centre, Institute of Clinical and Experimental Medicine, Prague (Czech Republic). Lipid concentrations in serum were measured by conventional enzymatic method. Subjects were classified into three groups according to their total cholesterol level: normal (<5.2 mmol/L), intermediate (5.2–6.19 mmol/L), and increased level (equal 6.2 mmol/L or more). Concentration of glucose in capillary blood was determined by an individual glucometer “Glucotrend” [20]. Normal glucose level was defined as fasting glucose <5.55 mmo/L, intermediate as glucose level 5.55–6.98 mmol/L and increased as glucose level equal 6.99 mmol/L or more [6], [20].

### Variables determined using the questionnaire

The structured questionnaire included questions regarding the respondent's age, education, smoking status, alcohol consumption, physical activity, antihypertensive treatment, hypoglycemic therapy, lipid lowering treatment, and other factors. Education was classified into four education levels: primary, vocational, secondary or college and university. Smoking status was classified as never smokers, former smokers and current smokers (at least one cigarette per day). Alcohol consumption frequency was classified into six groups: never or former drinkers, drinkers less than once per month, 1–3 times per month, once per week, 2–3 times per week, several times per week or daily. Physical activity was determined by weekly hours of leisure activity in winter and summer (e.g. walking, moderate and hard work, gardening and other physical activities). The respondents were categorized into three groups according to their physical activity in leisure time: active (7 and more hours/week), intermediate (2.0–6.99 hours/week), and inactive (<2.0 hours/week).

### Healthy factors

Healthy factors measured at baseline include non-smoking, normal weight, normal arterial blood pressure, normal level of total serum cholesterol, normal physical activity and normal level of fasting glucose.

### Follow-up

Participants (aged 45–64) of the five surveys form the analytical cohort. Deaths among the participants between the baseline (of the relevant survey) until the end of 2011 were identified from the regional mortality register. Causes of death were coded by versions 9 and 10 of the International Classification of Diseases: CVD mortality included codes 390–458 of ICD-9 and I00-I99 – codes of ICD-10; deaths from CHD included codes 410–414 of ICD-9 and I20–I25 of ICD-10. During 1983–2011, there were 1,219 death cases from any cause, 589 deaths from CVD, and 342 deaths from CHD among persons free from CHD and stroke at baseline surveys. The mean duration of follow-up was 13.3 years.

### Statistical analysis

Descriptive statistics (prevalence rates, means and standard deviations (SD)) were calculated for variables in each survey. P<0.05 values were taken as statistically significant. Hazard ratio (HR) and 95% confidence intervals (CI) were estimated by the multivariate Cox proportional hazards regression for all-cause, CVD and CHD mortality separately for men and women. The model included age, survey year, education, alcohol consumption, antihypertensive treatment, hypoglycemic therapy, lipid lowering treatment, and all six risk factors (smoking habits, body weight, BP, total cholesterol, fasting glucose, physical activity). We also presented model including the number of cardiovascular health factors. The HR were calculated and compared to the average risk in the whole population using deviation from mean coding. The analytical sample was restricted to persons free from CHD and stroke at baseline and having all data for variables included into multivariate Cox models (n = 4,979). Data were analyzed with SPSS version 13.4 software for Windows.

## Results

Age-standardized means and the prevalence rates of cardiovascular factors by survey are presented in Table 1. All cohorts had similar age and gender distribution whereas the educational distribution changed over time. There were also some changes in cardiovascular risk profile. Mean fasting glucose increased from 4.61 (1.16) to 5.79 (1.17) mmol/L comparing 1983–1984 and 2006–2008 surveys samples (p<0.05), as did the proportion of responders with fasting glucose ≥6.99 mmol/l. The proportion of subjects in intermediate leisure physical activity group decreased during the study period (p<0.05) and proportion of subjects receiving antihypertensive treatment during the same period increased (p = 0.001). The proportion of subjects with 6 healthy factors was higher in 2006–2008 than in 1983–1984 (p = 0.09). On the other hand, during 25 year period, the levels of total serum cholesterol, smoking habits, and blood pressure did not change.

**Table 1:** Characteristics of population and prevalence (%) of risk factors in urban population aged 45–64 years in 1983–1984 to 2006–2008.

Risk factors	1983–1984	1986–1987	1992–1993	2001–2002	2006–2008	P for
	(n = 1682)	(n = 1192)	(n = 848)	(n = 1001)	(n = 4486)	trend
**Mean age, **years (SD)	54.6 (5.40)	53.9 (5.64)	54.4 (5.76)	54.2 (5.57)	56.1 (5.53)	0.30
**Sex**						
Men	46.5	50.7	49.2	43.4	44.8	0.20
Women	53.5	49.3	50.8	56.6	55.5	0.20
**Smoking status**						
Never	67.5	69.7	71.4	64.5	57.9	0.12
Former	14.9	11.5	14.2	14.7	17.7	0.20
Current	17.6	18.8	14.4	20.8	24.4	0.19
**Leisure physical activity**						
Active	67.5	69.7	83.9	78.6	87.9	0.07
Intermediate	24.8	27.3	14.9	15.0	9.9	**<0.05**
Inactive	7.7	4.9	1.2	6.3	2.2	0.47
**BMI, kg/m^2^**						
<25.0	17.6	19.0	27.2	23.8	22.6	0.33
25.0–29.9	41.1	41.7	42.5	37.2	39.4	0.10
≥30.0	41.5	39.3	30.4	39.0	38.0	0.80
Mean BMI, kg/m^2^ (SD)	29.5 (5.40)	29.2 (4.79)	28.3 (4.71)	29.0 (5.78)	29.0 (5.34)	0.66
**Total serum cholesterol,** mmol/L						
<5.2	18.6	16.6	22.1	20.2	25.2	0.12
5.2–6.19	37.2	31.6	31.4	31.2	35.7	0.84
>6.2	44.4	51.8	46.6	48.6	39.1	0.42
**Mean total cholesterol**, mmol/L (SD)	6.14 (1.19)	6.31 (1.18)	6.26 (1.35)	6.33 (1.34)	5.98 (1.13)	0.56
**Lipid-lowering treatment**	ND	ND	0.6	1.3	7.7	0.11
**Blood pressure**, mm Hg						
<120/80 (untreated)	8.1	13.6	8.9	14.6	11.9	0.41
120–139/80–89 or treated to <120/80	30.9	33.4	32.6	29.7	25.8	0.11
≥140/≥90	61.0	53.0	58.5	55.7	62.3	0.67
**Mean BP**, mmHg (SD**)**						
Systolic	143.7 (23.8)	139.4 (22.2)	142.2 (22.5)	139.3 (23.2)	138.6 (21.4)	0.18
Diastolic	88.3 (11.8)	86.6 (12.8)	89.1 (11.5)	86.3 (12.4)	90.5 (12.6)	0.59
**Antihypertensive treatment**	19.1	21.1	26.3	32.8	33.2	**0.001**
**Fasting blood glucose**, mmol/L						
<5.55	83.7	69.3	52.9	60.6	44.9	0.08
5.55–≤6.99	14.4	26.9	42.5	33.8	46.6	0.10
>6.99	1.8	3.8	4.6	5.5	8.5	**<0.05**
**Mean fasting glucose**, mmol/L (SD)	4.61 (1.16)	5.09 (0.99)	5.39 (1.48)	5.62 (1.53)	5.79 (1.17)	**<0.05**
**Hypoglycemic therapy**	1.6	2.2	1.6	1.9	4.5	0.21
**Education**						
Primary	26.5	18.5	12.1	1.5	2.0	**<0.01**
Unfinished secondary	25.9	27.4	22.1	9.7	31.8	0.76
Secondary or college	30.0	31.6	39.7	59.4	31.6	0.43
University	17.6	22.5	26.1	29.4	34.6	**<0.01**
**Number of healthy levels of risk factors**						
0	2.2	1.8	1.1	2.3	2.3	0.59
1	14.1	12.8	11.9	12.3	15.6	0.64
2	31.9	31.3	33.4	31.7	34.9	0.32
3	33.9	38.4	31.6	33.3	29.7	0.21
4	14.9	11.7	16.9	15.5	13.2	0.91
5	2.7	3.5	4.7	4.3	3.7	0.37
6	0.2	0.5	0.4	0.6	0.6	0.09

BMI – body mass index. ND – no data. SD – standard deviation.

A greater number of healthy levels of cardiovascular risk factors was related to lower all-cause mortality (all-cause mortality rate among subjects with 0 health factors compared to subjects having 6 health factors was 21.2% and 5.0% respectively) (Table S1). After multivariate adjustment for age, education, alcohol intake frequency, survey, antihypertensive and lipid-lowering treatment, and hypoglycemic therapy being never smoking and being at active level of leisure physical activity, having BMI 25.0–29.9 kg/m^2^ among men and being at active level of leisure physical activity, having intermediate BP level, fasting glucose level less 5.55 mmol/L among women were each associated with a significantly lower risk of all-cause mortality (Table 2). The only factor that was not associated with mortality in the expected fashion was total cholesterol among men; compared to the average risk in the whole population using deviation from mean coding, participants with cholesterol level <5.2 mmol/L the fully adjusted HR for all-cause mortality was significantly higher (HR 1.19, 1.05–1.36). A similar pattern was observed for CVD mortality (Table 3) and CHD mortality (table 4) among women. The tendency of lower but not statistically significant all-cause, CVD and CHD risk of the intermediate cholesterol category was determined both among men and women.

**Table 2:** Adjusted hazard ratio (HR) of cardiovascular health factors and risk of all-cause mortality.

			HR (95% CI)			
Cardiovascular health factor	Men			Women		
		Number of deaths/number at risk	P value		Number of deaths/number at risk	P value
*Smoking status*						
Current	**1.43 (1.26–1.62)**	50/839	**<0.001**	1.27 (0.83–1.93)	3/257	0.27
Former	0.93 (0.81–1.08)	39/593	0.33	0.98 (0.61–1.59)	3/164	0.94
Never	**0.75 (0.66–0.86)**	45/878	**<0.001**	0.80 (0.60–1.08)	58/2158	0.15
*Leisure physical activity*						
Inactive	1.16 (0.90–1.50)	11/83	0.25	**1.81 (1.30–2.53)**	4/58	**0.001**
Intermediate	1.06 (0.89–1.25)	44/427	0.52	0.84 (0.66–1.06)	14/334	0.14
Active	**0.82 (0.70–0.96)**	79/1800	**0.011**	**0.66 (0.54–0.81)**	46/2187	**<0.001**
*BMI,* kg/m^2^						
≥30.0	1.03 (0.89–1.19)	50/628	0.68	1.09 (0.90–1.32)	34/1081	0.36
25.0–29.9	**0.84 (0.74–0.95)**	52/1076	**0.006**	0.88 (0.73–1.08)	21/915	0.23
<25.0	**1.15 (1.00–1.32)**	32/606	**0.044**	1.04 (0.81–1.33)	9/583	0.78
*Blood pressure*, mm Hg						
≥140/≥90	**1.20 (1.03–1.38)**	89/1419	**0.017**	1.22 (0.98–1.51)	48/1357	0.07
120–139/80–89 or treated to <120/80	0.93 (0.80–1.09)	37/671	0.36	**0.76 (0.61–0.96)**	14/793	**0.022**
<120/80 (untreated)	0.90 (0.73–1.11)	8/220	0.31	1.08 (0.80–1.44)	2/429	0.63
*Fasting glucose,* mmol/L						
>6.99	1.20 (0.91–1.58)	9/132	0.20	**1.61 (1.09–2.41)**	7/138	**0.018**
5.55–≤6.99	0.87 (0.73–1.04)	33/900	0.12	0.80 (0.62–1.02)	16/1000	0.08
<5.55	0.96 (0.81–1.14)	92/1278	0.65	**0.78 (0.61–0.999)**	41/1441	**0.049**
*Total serum cholesterol,* mmol/L						
≥6.2	0.90 (0.80–1.02)	54/862	0.11	1.02 (0.85–1.22)	35/1254	0.82
5.2–6.19	0.93 (0.82–1.06)	39/829	0.27	0.92 (0.75–1.13)	17/847	0.92
<5.2	**1.19 (1.05–1.36)**	41/619	**0.008**	1.06 (0.83–1.36)	12/478	0.63

Adjusted for age, education, alcohol intake frequency, study survey year, smoking status, physical activity, BMI, total cholesterol level, blood pressure, fasting glucose level, antihypertensive treatment, hypoglycemic therapy, lipid lowering treatment. CI – confidence interval, BMI – body mass index.

**Table 3:** Adjusted hazard ratio (HR) of cardiovascular health factors and risk of CVD mortality.

			HR (95% CI)			
Cardiovascular health factor	Men			Women		
		Number of deaths/number at risk	P value		Number of deaths/number at risk	P value
*Smoking status*						
Current	**1.50 (1.23–1.82)**	91/839	**<0.001**	1.17 (0.60–2.29)	6/257	0.65
Former	0.90 (0.73–1.11)	54/593	0.31	0.97 (0.47–2.04)	4/164	0.94
Never	**0.75 (0.61–0.91)**	75/878	**0.003**	0.88 (0.56–1.39)	115/2158	0.59
*Leisure physical activity*						
Inactive	1.33 (0.94–1.87)	17/83	0.11	1.58 (0.95–2.64)	8/58	0.08
Intermediate	1.01 (0.79–1.28)	65/427	0.96	0.80 (0.56–1.15)	29/334	0.23
Active	**0.75 (0.60–0.94)**	138/1800	**0.012**	0.79(0.58–1.07)	88/2187	0.13
*BMI,* kg/m^2^						
≥30.0	**1.36 (1.11–1.67)**	76/628	**0.003**	0.93 (0.71–1.22)	70/1081	0.60
25.0–29.9	0.84 (0.70–1.02)	94/1076	0.07	0.95 (0.72–1.26)	37/915	0.74
<25.0	0.87 (0.70–1.09)	50/606	0.24	1.13 (0.79–1.62)	18/583	0.50
*Blood pressure*, mm Hg						
≥140/≥90	**1.44 (1.14–1.81)**	150/1419	**0.002**	**1.61 (1.13–2.29)**	97/1357	**0.008**
120–139/80–89 or treated to <120/80	0.87 (0.67–1.11)	54/671	0.26	0.74 (0.50–1.09)	21/793	0.13
<120/80 (untreated)	0.80 (0.56–1.14)	16/220	0.22	0.84 (0.49–1.45)	7/429	0.53
*Fasting glucose,* mmol/L						
>6.99	1.34 (0.90–2.02)	14/132	0.15	**2.27 (1.32–3.90)**	11/138	**0.003**
5.55–≤6.99	**0.75 (0.57–0.98)**	57/900	**0.034**	**0.68 (0.47–0.98)**	30/1000	**0.039**
<5.55	0.99 (0.77–1.27)	149/1278	0.95	**0.65 (0.46–0.92)**	84/1441	**0.015**
*Total serum cholesterol,* mmol/L						
≥6.2	1.03 (0.86–1.24)	94/862	0.76	0.93 (0.72–1.20)	75/1254	0.57
5.2–6.19	0.82 (0.67–1.01)	59/829	0.06	0.74 (0.55–1.00)	29/847	0.052
<5.2	1.18 (0.97–1.44)	67/619	0.10	**1.45 (1.04–2.02)**	21/478	**0.028**

Adjusted for age, education, alcohol intake frequency, study survey year, smoking status, physical activity, body mass index, total cholesterol level, blood pressure, fasting glucose level, antihypertensive treatment, hypoglycemic therapy, lipid lowering treatment. CI – confidence interval, CVD – cardiovascular, BMI – body mass index.

**Table 4:** Adjusted hazard ratio (HR) of cardiovascular health factors and risk of CHD mortality.

			HR (95% CI)			
Cardiovascular health factor	Men			Women		
		Number of deaths/number at risk	P value		Number of deaths/number at risk	P value
*Smoking status*						
Current	**1.32 (1.03–1.69)**	50/839	**0.031**	1.10 (0.44–2.71)	3/257	0.84
Former	1.04 (0.81–1.35)	39/593	0.74	1.43 (0.58–3.51)	3/164	0.43
Never	**0.73 (0.57–0.93)**	45/878	**0.013**	0.64 (0.35–1.15)	58/2158	0.64
*Leisure physical activity*						
Inactive	1.38 (0.89–2.13)	11/83	0.15	1.47 (0.71–3.05)	4/58	0.31
Intermediate	1.05 (0.78–1.42)	44/427	0.74	0.83 (0.50–1.39)	14/334	0.83
Active	**0.69 (0.52–0.92)**	79/1800	**0.010**	0.82 (0.52–1.28)	46/2187	0.82
*BMI,* kg/m^2^						
≥30.0	**1.40 (1.09–1.81)**	50/628	**0.009**	0.87 (0.60–1.26)	34/1081	0.46
25.0–29.9	**0.76 (0.60–0.97)**	52/1076	**0.03**	1.09 (0.73–1.61)	21/915	0.68
<25.0	0.94 (0.70–1.24)	32/606	0.64	1.06 (0.64–1.76)	9/583	0.81
*Blood pressure*, mm Hg						
≥140/≥90	**1.38 (1.00–1.89)**	89/1419	**0.048**	1.62 (0.91–2.89)	48/1357	0.10
120–139/80–89 or treated to <120/80	1.00 (0.72–1.39)	37/671	0.99	1.22 (0.66–2.24)	14/793	0.53
<120/80 (untreated)	0.73 (0.44–1.20)	8/220	0.21	0.51 (0.19–1.37)	2/429	0.18
*Fasting glucose,* mmol/L						
>6.99	1.47 (0.88–2.46)	9/132	0.14	**2.75 (1.35–5.64)**	7/138	**0.006**
5.55–≤6.99	**0.71 (0.50–0.99)**	33/900	**0.046**	0.63 (0.38–1.03)	16/1000	0.07
<5.55	0.96 (0.70–1.32)	92/1278	0.81	**0.58 (0.36–0.93)**	41/1441	**0.025**
*Total serum cholesterol,* mmol/L						
≥6.2	0.99 (0.78–1.25)	54/862	0.91	0.75 (0.54–1.21)	35/1254	0.12
5.2–6.19	0.86 (0.67–1.11)	39/829	0.25	0.81 (0.54–1.21)	17/847	0.31
<5.2	1.18 (0.91–1.52)	41/619	0.21	**1.64 (1.04–2.59)**	12/478	**0.033**

Adjusted for age, education, alcohol intake frequency, study survey year, smoking status, physical activity, body mass index, total cholesterol level, blood pressure, fasting glucose level, antihypertensive treatment, hypoglycemic therapy, lipid lowering treatment. CHD – coronary heart disease, CI – confidence interval, BMI – body mass index.

Table 5 shows the adjusted HR for all-cause, CVD and CHD mortality among men, women, and in both sexes combined by the number of healthy levels of cardiovascular risk factors, comparing those with average risk in the whole population. Having favourable levels on a greater number of health factors was associated with a lower risk of all-cause, CVD and CHD mortality, although the trend was stronger in men and more pronounced for CVD mortality.

**Table 5:** Adjusted* hazard ratios (HR) of mortality by the number of cardiovascular health factors.

					Sex				
Number of healthy levels of risk factors		Men			Women			Men and women	
	HR (95% CI)	Number of deaths/number at risk	P value	HR (95% CI)	Number of deaths/number at risk	P value	HR (95% CI)	Number of deaths/number at risk	P value
***All-cause mortality***									
0–1	1.04 (0.85–1.27)	104/540	0.69	**1.55 (1.08**–**2.22)**	29/223	**0.018**	1.14 (0.96–1.36)	133/763	0.14
2	1.04 (0.87–1.25)	175/804	0.67	1.12 (0.84–1.50)	83/809	0.44	1.08 (0.93–1.26)	258/1613	0.31
3–4	1.04 (0.87–1.24)	195/894	0.70	0.98 (0.74–1.29)	140/1370	0.89	1.03 (0.89–1.20)	335/2264	0.66
5–6	0.89 (0.60–1.32)	15/72	0.56	0.59 (0.30–1.15)	5/177	0.12	0.78 (0.56–1.10)	20/249	0.15
***CVD mortality***									
0–1	1.50 (0.99–2.27)	50/540	0.054	1.61 (0.83–3.11)	12/223	0.16	**1.50 (1.06–2.12)**	62/763	**0.023**
2	**1.50 (1.02–2.23)**	86/804	**0.042**	1.29 (0.73–2.27)	38/809	0.39	**1.42 (1.03–1.96)**	124/1613	**0.032**
3–4	1.30 (0.88–1.92)	82/894	0.19	1.28 (0.74–2.22)	74/1370	0.37	1.33 (0.97–1.83)	156/2264	0.08
5–6	**0.34 (0.12–0.97)**	2/72	**0.044**	0.38 (0.09–1.67)	1/177	0.20	**0.35 (0.15–0.83)**	3/249	**0.017**
***CHD mortality***									
0–1	1.20 (0.93–1.55)	36/540	0.17	1.38 (0.84–2.29)	8/223	0.21	**1.35 (1.07–1.69)**	44/763	
2	0.88 (0.69–1.12)	45/804	0.29	0.83 (0.55–1.24)	18/809	0.36	0.85 (0.69–1.04)	63/1613	0.12
3–4	0.95 (0.75–1.20)	53/894	0.67	0.87 (0.61–1.25)	38/1370	0.46	0.88 (0.72–1.06)	91/2264	0.17
5–6	–	0/72	–	–	0/177	–	–	0/249	–

* - adjusted for age, education, alcohol intake frequency, study survey year, and sex (in the models among men and women in one model). CI – confidence interval, CHD – coronary heart disease, CVD – cardiovascular.

## Discussion

To our knowledge, the present study is the first prospective investigation of the prevalence, trends and prognostic impact of healthy levels of cardiovascular risk factors on all-cause, CVD and CHD mortality in the Baltics. We found that only about 1% of middle-aged urban adults in Kaunas had a low-risk profile. This indicates the large potential for preventing cardiovascular and other non-communicable diseases that still remains to be implemented.

After the American Heart Association (AHA) defined the concept of cardiovascular health in the 2020 Impact Goal [21], several studies estimated the profile of ideal or low-risk cardiovascular health using the AHA criteria in the US and China [6], [9], [10]. The impact of favourable cardiovascular risk profile on CVD incidence and mortality using definitions to AHA have been previously evaluated in the US, Italy, Japan and other countries [7], [8], [23], [24].

Our data suggest very few changes in the prevalence, distribution and mean levels of 6 cardiovascular health factors during 25 years in this urban adult population. Some of these changes were unfavourable (e.g. the increase in the prevalence of unhealthy level of fasting glucose). On the other hand, there were also positive changes, reflected by the increase of the proportion of participants with 6 healthy levels of cardiovascular risk factors. In comparison, among the National Health and Nutrition Examination Survey (NHANES) III participants in the US, from 7 cardiovascular health metrics using AHA definition, for smoking showed favourable changes: proportion of never-smokers significantly increased from 1988–1994 to 2005–2010 [6]. For other cardiovascular health metrics, unfavourable (BMI, fasting glucose, and healthy diet status) or no changes (blood pressure, physical activity, and total serum cholesterol) have been detected. More favourable trends in the prevalence of low risk factors were observed among 25–74 year-aged participants in four NHAHES surveys (from 1971–1975 to 1999–2004). The prevalence of 3 low risk factors (out of 5) significantly improved (not smoking, low total serum cholesterol and favourable BP) but adverse trends have been observed for prevalence of BMI <25.0 kg/m*^2^* and not having diagnosed diabetes mellitus [22].

Considering the prevalence of ideal levels of cardiovascular risk factors, our findings indicate that the distribution of cardiovascular health metrics is different between Lithuania and other countries. Around two thirds (56.5% to 70.8%) of participants in our study were never smokers; this is significantly higher than the proportion of participants in other studies in US and Italy [24]–[26] but lower when compared to participants in Disease Risk Evaluation and Health Management (DREHM) study in China [6]. This, however, is mainly driven by low prevalence of smoking in women in Lithuania. The prevalence of ideal BMI, total serum cholesterol, BP, and fasting glucose were lower in our study than in the above studies. Only 8.6 to 15.6% of participants met the ideal level of BP after adjusting for age in our study. Furthermore, recently published findings from the MONICA study showed low hypertension control in men (3.6% in 1983–1984 and 12.6% in 2001–2002) and women (3.5% and 16.6% respectively) [5]. The prevalence of ideal level of physical activity was high (67.7 and 88.0%), compared to other studies [6], [27]. These differences partially could be explained by the fact that The International Physical Activity Questionnaire has been used for measuring of physical activity only in some surveys of our dataset. Therefore we could measure only the mean time a week spent during leisure for walking, vigorous-intensity and moderate-intensity exercise and hard work, gardening and other physical activities and we used our own definition of ideal, intermediate and unhealthy level of physical activity which differ from the AHA definition and criteria of physical inactivity used in the mentioned above studies.

In the prospective analyses, ideal or intermediate levels of most cardiovascular health factors were associated with significantly lower all-cause, CVD and CHD mortality, and many of these associations followed a dose-response pattern. These findings are consistent with previous findings in other cohorts [25], [28]–[30]. Interestingly, we found that total cholesterol concentrations <5.2 mmol/L were associated with increased higher risk of mortality when compared with the average risk in the whole population, although the NHANES III study also reported such an association – the adjusted hazard ratio of all-cause mortality for total serum cholesterol of <200 mg/dL (<5.2 mmol/L) vs.≥200 mg/dL (>5.2 mmol/L) was independently associated with a significantly higher risk of all-cause mortality (HR = 1.28 (95% CI 1.15–1.42) [6]. A meta-analysis of individual data from 61 prospective studies indicated, that total cholesterol was positively associated with ischaemic heart disease mortality in middle and old age groups, however the proportional risk reduction decrease with increasing BP, since the absolute effects of cholesterol and BP are approximately additive [31]. The absence of an independent positive association of total cholesterol with all-cause and CVD mortality in our longitudinal study, is unexplained, and invites for further research.

Most prospective studies relating ideal cardiovascular health or positive health factors observed an inverse relationship between the number of ideal (positive) health factors and mortality from all causes, CVD [6], [25], [30] and sudden cardiac death [32]. Similar findings were reported for the incidence of CHD [26], CVD [10] and stroke [24], [27]. This is consistent with our results: larger numbers of positive health factors were associated with lower CVD risk, although the association was stronger among men.

To date, no studies on this important topic were reported from Central and Eastern Europe where both the prevalence of cardiovascular risk factors and CVD mortality rates are high. The strength of our study includes the prospective cohort design, which makes selection and information bias unlikely. Numerator-denominator bias is minimized trough linkage of the survey cohorts with mortality register; the register is complete and it is very unlikely that it would miss more than a handful of deaths. In addition, we controlled for a range of potential confounding variables, including age, education, alcohol intake frequency and study survey year. An important advantage is the high comparability with other studies because definitions of most cardiovascular factors were identical to those recommended by AHA or those used in other cohort studies elsewhere.

However, the present study has several limitations. Our study involved only one assessment of the included healthy cardiovascular factors. Thus, because participants could have misreported or changed their lifestyle, there is a potential for exposure misclassification, which may have affected our risk estimates, most likely towards under-estimation. Nevertheless, most cardiovascular risk factors included in the analyses were found to be predictive for both all-cause and CVD mortality. Some of the earlier cohort studies found that, although many variables and risk factors changed over time, the baseline survey data remained predictive for mortality [33]. The next problem is that physical activity and nutritional habits in the surveys of forming our study were assessed using different questionnaires, we were unable to evaluate physical activity and diet score as recommended by AHA and we had to use our own ad hoc definitions. Another limitation is that capillary blood glucose was measured in all five general population surveys. It is likely that our measures were less reliable and less sensitive to detect adverse effects on mortality. Finally, the present study did not examine the national samples, but it rather included random population samples in one urban setting.

## Conclusions

The prevalence of ideal levels of all 6 easily measurable cardiovascular health factors in this Lithuanian general population sample was low. We observed an inverse association of most healthy levels of cardiovascular risk factors with risk of all-cause and CVD mortality, and greater number of cardiovascular health factors was significantly associated with lower risk of CVD mortality among men. The strategic priority for public health is to increase the proportion of the population with ideal levels of cardiovascular health factors in order to reduce CVD mortality.

## Supporting Information

Table S1 All-cause, CVD, and CHD mortality* according to number of cardiovascular health factors.
(DOCX)
