# Supplementary material for: Health Factors and Risk of All-Cause, Cardiovascular, and Coronary Heart Disease Mortality: Findings from the MONICA and HAPIEE Studies in Lithuania
Source: PLoS One. 2014 Dec 5;9(12):e114283. doi: 10.1371/journal.pone.0114283 (PMC4257606; doi:10.1371/journal.pone.0114283)
Supplement: Table S1 — All-cause, CVD, and CHD mortality* according to number of cardiovascular health factors. (DOCX) [file pone.0114283.s001.docx]

**Supplementary Table. A**ll-cause, CVD, and CHD mortality* according to number of cardiovascular health factors

|  |  |  |  |  |  |  | **Sex** |  |  |  |  |  |
| --- | --- | --- | --- | --- | --- | --- | --- | --- | --- | --- | --- | --- |
| **Number of healthy levels of risk factors** |  |  | **Men (n=2339)** |  |  |  | **Women (n=2640)** |  |  |  | **Men and women (n=4979)** |  |
|  | **Alive n (%)** | **All-cause mortality n (%)** | **CVD mortality n (%)** | **CHD mortality n (%)** | **Alive n (%)** | **All-cause mortality n (%)** | **CVD mortality n (%)** | **CHD mortality n (%)** | **Alive n (%)** | **All-cause mortality n (%)** | **CVD mortality n (%)** | **CHD mortality n (%)** |
| 0 | 55 (78.6) | 15 (21.4) | 4 (5.7) | 2 (2.9) | 12 (80.0) | 3 (20.0) | 0 (0.0) | 0 (0.0) | 67 (78.8) | 18 (21.2) | 4 (4.7) | 2 (2.4) |
| 1 | 389 (82.9) | 80 (17.1) | 42 (9.0) | 31 (6.6) | 184 (88.9) | 23 (11.1) | 10 (4.8) | 6 (2.9) | 573 (84.8) | 103 (15.2) | 52 (7.7) | 37 (5.5) |
| 2 | 651 (79.4) | 169 (20.6) | 81 (9.9 | 42 (5.1) | 723 (90.6) | 75 (9.4) | 34 (4.3) | 16 (2.0) | 1375 (84.8) | 246 (15.2) | 115 (7.1) | 58 (3.6) |
| 3 | 498 (79.7) | 127 (20.3) | 54 (8.6) | 34 (5.4) | 861 (89.3) | 103 (10.7) | 53 (5.5) | 26 (2.7) | 1360 (85.5) | 230 (14.5) | 108 (6.8) | 60 (3.8) |
| 4 | 226 (80.1) | 56 (19.9) | 20 (7.1) | 13 (4.6) | 425 (92.6) | 34 (7.4) | 16 (3.5) | 9 (2.0) | 651 (87.6) | 92 (12.4) | 37 (5.0) | 23 (3.1) |
| 5 | 51 (79.7) | 13 (20.3) | 2 (3.1) | 0 (0.0) | 161 (97.0) | 5 (3.0) | 1 (0.6) | 0 (0.0) | 212 (92.6) | 17 (7.4) | 2 (0.9) | 0 (0.0) |
| 6 | 7 (77.8) | 2 (22.2) | 0 (0.0) | 0 (0.0) | 31 (100.0) | 0 (0.0) | 0 (0.0) | 0 (0.0) | 38 (95.0) | 2 (5.0) | 0 (0.0) | 0 (0.0) |
| Total | 1877 (80.2) | 462 (19.8) | 203 (8.7) | 122 (5.2) | 2397 (90.8) | 243 (9.2) | 114 (4.3) | 57 (2.2) | 4276 (85.8) | 708 (14.2) | 318 (6.4) | 180 (3.6) |

*age- and sex-standardized. CHD – coronary heart disease, CVD – cardiovascular.
